# Randomised controlled trial and economic evaluation of the ‘Families for Health’ programme to reduce obesity in children

**DOI:** 10.1136/archdischild-2016-311514

**Published:** 2016-12-21

**Authors:** Wendy Robertson, Joanna Fleming, Atiya Kamal, Thomas Hamborg, Kamran A Khan, Frances Griffiths, Sarah Stewart-Brown, Nigel Stallard, Stavros Petrou, Douglas Simkiss, Elizabeth Harrison, Sung Wook Kim, Margaret Thorogood

**Affiliations:** Division of Health Sciences, Warwick Medical School, University of Warwick, Coventry, UK

**Keywords:** Obesity, Nutrition, Evidence Based Medicine, Parenting

## Abstract

**Objective:**

Evaluating effectiveness and cost-effectiveness of ‘Families for Health V2′ (FFH) compared with usual care (UC).

**Design:**

Multicentre randomised controlled trial (RCT) (investigators blinded, families unblinded) and economic evaluation. Stratified randomisation by family; target of 120 families.

**Setting:**

Three National Health Service Primary Care Trusts in West Midlands, England.

**Participants:**

Overweight or obese (≥91st or ≥98th centile body mass index (BMI)) children aged 6–11 years and their parents/carers, recruited March 2012–February 2014.

**Interventions:**

FFH; a 10-week community-based family programme addressing parenting, lifestyle change and social and emotional development. UC; usual support for childhood obesity at each site.

**Main outcome measures:**

Primary outcomes were 12-months change in children's BMI z-score and incremental cost per quality-adjusted life-year gained (QALY). Secondary outcomes included changes in children's physical activity, fruit and vegetable consumption and quality of life, parents' BMI and mental well-being, family eating/activity, parent-child relationships and parenting style.

**Results:**

115 families (128 children) were randomised to FFH (n=56) or UC (n=59). There was no significant difference in BMI z-score 12-months change (0.114, 95% CI −0.001 to 0.229, p=0.053; p=0.026 in favour of UC with missing value multiple imputation). One secondary outcome, change in children's waist z-score, was significantly different between groups in favour of UC (0.15, 95% CI 0.00 to 0.29). Economic evaluation showed that mean costs were significantly higher for FFH than UC (£998 vs £548, p<0.001). Mean incremental cost-effectiveness of FFH was estimated at £552 175 per QALY.

**Conclusions:**

FFH was neither effective nor cost-effective for the management of obesity compared with UC.

**Trial registration number:**

ISRCTN45032201.

**What is already known on this topic?**

- Childhood obesity represents a major public health burden, with a third of children aged 10–11 years in England being either overweight or obese. Effective treatment interventions are needed.
- The Families for Health programme was a promising intervention for the management of childhood obesity in a pilot, focusing on parenting skills, social and emotional development as well as lifestyle.

**What this study adds?**

- There was no significant difference in change in BMI z-score at 12-months with Families for Health compared to usual care.
- Families for Health was significantly more costly than usual care.
- The Families for Health programme is neither effective nor cost-effective for the treatment of obesity in children aged 6 to 11.

## Introduction

Childhood obesity represents a major public health burden. Although trends may now be reversing in England,^1^ prevalence remains high. In 2014–2015, 33.2% of children in year 6 (10–11 years) were either overweight or obese.^2^ Childhood overweight and obesity have been linked to immediate and long-term physiological and psychological health risks,^3^ ^4^ including type 2 diabetes, hypertension and coronary heart disease in adulthood.^5^

A review of 64 randomised controlled trials (RCTs) of interventions for childhood obesity concluded that family-based interventions combining dietary, physical activity and behavioural components produce significant improvement.^6^ In children under 12 years, involving parents was useful. Family-based interventions for childhood obesity are being offered, but they rarely cover parenting skills.

‘Families for Health’ (FFH) is a manualised group-based family intervention for overweight or obese children aged 6–11 years. The programme places more emphasis on parenting skills, relationship skills and emotional and social development than other UK interventions. A pre-post pilot of 27 children showed mean reductions in body mass index (BMI) z-scores sustained at 9 months (−0.21, 95% CI −0.35 to −0.07, p=0.007) and 2 years (−0.23, 95% CI −0.42 to 0.03, p=0.027),^7^ ^8^ encouraging further evaluation. This study examines the effectiveness and cost-effectiveness of FFH.

## Methods

### Study design

In a multicentre RCT in overweight or obese children aged 6–11 years, with parallel economic and process evaluations, families were randomised to FFH (target 60 families) or usual care (UC) (target 60 families) (ISRCTN45032201). Randomisation, via a central telephone registration and randomisation service of Warwick Clinical Trials Unit, was stratified by locality using biased coin (p=2/3) minimisation to ensure approximately equal-sized arms. Families could not be blinded to treatment allocation, but every effort was made to ensure that allocation remained unknown to researchers.

### Participants

This trial took place between March 2012 and March 2015 in three defined areas within the West Midlands, UK, reflecting the varied demographics within the region. Sites A and C were relatively more deprived, whereas site B was less deprived (IMD 2015 rank out of 326 Local Authority Districts, where one is the most deprived: site A 55, site B 249, site C 14).^9^ Sites A and C also had greater ethnic diversity (% white ethnicity: site A 73.8%, site B 92.6%, site C 67.9%).^10^

Eligible families had an overweight (≥91st centile for BMI) or obese (≥98th centile for BMI) child aged 6–11 years, based on the UK 1990 definition^11^; and at least one parent or guardian willing to take part. Families were excluded if parent or child had insufficient command of English; the child had recognised medical cause of obesity or was unable to participate due to severe learning difficulties and/or behavioural problems. We aimed to recruit 40 families from each of three sites, using active and passive recruitment methods.^12^ Active recruitment methods were via letters to families with an eligible child identified by the National Child Measurement Programme and by referrals from healthcare professionals, including dietitians and general practitioners (GPs). Passive recruitment methods were via the local media (newspapers and radio); flyers and posters at schools, GP surgeries and other community venues and attending public events.

### The intervention: families for health

The FFH intervention (V1) was developed by Candida Hunt and the University of Warwick team (SS-B, WR). Following evaluation in the pilot,^7^ ^8^ the programme underwent minor modifications and it is FFH V2 that was delivered in this RCT. The changes in V2, implemented based on parents' feedback, were the reduction in length from 12 to 10 weeks, the addition of two follow-up sessions, enhanced information on healthy eating and the distribution of pedometers. The FFH V2 manualised programme comprises 10 weekly 2½-hour sessions, with children and parents from 8 to 12 families attending parallel groups. The programme combines information on parenting skills, social and emotional development as well as healthy eating including portion size and physical activity. The plan was to run six FFH courses (two in each site). Parenting components, based on the Nurturing Programme from Family Links,^13^ aimed to increase parental capacity to implement and maintain lifestyle changes. Further details of the FFH intervention are available.^14^

Four facilitators, as pairs in the children's and parents' groups, ran each programme following a 4-day Family Links training course. Facilitators were selected for their personal attributes, including previous relevant experience. Professional backgrounds included community nursing, teaching, youth work, leisure services and nutritionists. We aimed to assign families to groups within 3 months of randomisation, and invited both parents and all overweight and non-overweight siblings in the target age range. Each group ran on a Saturday in a leisure/community centre. Additional sessions were planned for 1 month and 3 months post-intervention.

### Usual care control group

Families assigned to UC were offered ‘One Body One Life’,^15^ a group-based family intervention in site A, Change4Life advisors offering one-to-one support in site B and either (1) a two-step programme, MEND^16^ and Choose It, with taster sessions for physical activity, healthy eating, or (2) Weight Watchers for young people aged 10+ years or (3) referral to the school nurse for children aged 6–9 years in site C. Further details of the UC interventions are available.^14^

### Outcome measures

We collected outcome measures at home visits at baseline, 3 months (or end of FFH programme) and 12 months post-randomisation.

#### Demographic characteristics

At baseline parents completed a brief demographic questionnaire. Families' socioeconomic status (SES) was recorded using the National Statistics Socioeconomic Classification based on parental employment.^17^

#### Anthropometric measurements

The primary outcome was change in children's BMI z-score from baseline to 12 months. Weight was measured using the Tanita body composition analyser (BC-420S MA), which also provided an indirect measure of percentage body fat.^18^ Height was measured by a Leicester stadiometer. BMI (kg/m^2^) and waist circumference (with a Seca 200 tape) were converted into SD (z) scores from the UK reference curves.^11^ ^19^ ^20^ Parent's height, weight, BMI and percentage body fat were recorded.

#### Behavioural/lifestyle measurements

Children were asked to wear an accelerometer (Actigraph GT3X, Penascola, Florida, USA) for 7 consecutive days at baseline and 12-month follow-up. Data were analysed using Actilife 6 Data Analysis Software, using Evenson's activity count cut-points for physical activity intensities.^21^ We defined a complete day of data as ≥8 hours, after excluding any periods of ≥60 consecutive minutes of zero counts (non-wear time). Records were included in the analysis if at least 3 complete days of data were available at baseline and 12 months. Mean daily time in moderate and vigorous physical activity, sedentary time, accelerometer counts per minute and daily step count were calculated.

Children completed a 24-hour recall using the ‘Day in the Life Questionnaire’, which is validated for fruit and vegetable consumption.^22^ Eating and activity behaviour in the family was assessed using an Anglicised version of the Family Eating and Activity Habits Questionnaire (FEAHQ).^23^

#### Psychosocial measurements

Children's health-related quality of life was measured using the Pediatric Quality-of-Life Inventory (PedsQL) V.4.0 (UK) for ages 8–12 years.^24^ Children completed the 23-item self-report version and parents completed the parent-proxy version. Parental mental well-being was measured using the 14-item Warwick-Edinburgh Mental Well-Being Scale.^25^

The quality of parent-child relationships was measured using the parent-completed 15-item version of the Child-Parent Relationship Scale (CPRS).^26^ Parenting style was scored as authoritative, authoritarian or permissive parenting using the 32-item Parenting Styles and Dimensions Questionnaire.^27^

### Sample size calculation

Informed by the pilot,^7^ we based power calculations on a BMI z-score residual SD of 0.22, a SD of random family effects of 0.14, an intervention group intracluster correlation of 0.1, a two-sided significance of 5% and an estimate of 60% of participating families having one overweight/obese child and 40% having two. Allowing for clustering by family and for group effects in the intervention arm, 6 groups of 10 families (60 families) in the intervention arm and 60 families in the control arm provided power of 94% to detect an intervention effect of 0.2 in BMI z-score. If 30% of families dropped out, the study would retain power of 88%.

### Statistical methods

For child outcome measures, linear mixed models with a random family effect were fitted to account for clustering. After approval from the Trial Steering Committee, we did not account for delivery group clustering in the FFH arm as analyses showed no evidence of clustering. Separate models were fitted for differences between baseline and 3-month follow-up (end of FFH programme) and baseline and 12-month follow-up. Models were adjusted for baseline values of outcomes, gender and family-level ‘locality’ as fixed effects as specified in the Statistical Analysis Plan. Primary analyses were conducted on trial participants with complete relevant data. A preplanned secondary analysis was also conducted with missing values imputed using multiple imputation with fully conditional specification regression.^28^

We summarised outcomes by trial allocation and follow-up period using means, SDs and CIs for continuous variables and absolute numbers, percentages and CIs for categorical variables. Generally, one parent per family provided data, and parent outcomes were compared using t-tests and χ^2^ tests.

All analyses were performed on an intention-to-treat basis, except where clearly stated, and conducted using SAS V.9.4 TSL1M2.

### Economic evaluation

We conducted a within-trial economic evaluation from a UK National Health Service (NHS) and personal social services perspective.^29^ A comprehensive strategy was adopted to estimate incremental costs associated with the programme. Resource use questions completed by parents at each time-point provided profiles of hospital and community health and social services received by each child and broader service utilisation including educational support, family expenditures and parental lost productivity attributable to the child's health status. Unit costs (£, 2013–2014 prices) were collected from national sources in accordance with guidelines and attached to resource use.^29^ Health utilities generated from EuroQol Five Dimensions Questionnaire Youth Version (EQ-5D-Y) responses,^30^ ^31^ obtained from parents and children at each time point, were used to estimate quality-adjusted life year (QALY) profiles for each child, calculated as area under the baseline-adjusted utility curve, assuming linear interpolation between utility measurements. We report cost-effectiveness results as incremental cost-effectiveness ratios (ICERs), calculated as the difference in mean costs divided by the difference in mean outcomes (QALYs or change in BMI z-score between baseline and 12 months) between the trial comparators. The non-parametric bootstrap method was used to construct cost-effectiveness acceptability curves at alternative cost-effectiveness thresholds relevant to decision-makers. Secondary analyses adopted a wider societal perspective for economic costs. Sensitivity analyses were undertaken to assess the impact of uncertainty surrounding aspects of the economic evaluation, while subgroup analyses were conducted for the main cost-effectiveness results to explore the effects of trial population heterogeneity. Further details are reported in web appendix 1.

10.1136/annrheumdis-2016-210131.supp1 supplementary web appendix

### Process evaluation

We examined reach of the intervention, fidelity of delivery, dose delivered and received,^32^ and perceived impact of the intervention through observation, collection of trial process data, focus groups with facilitators and interviews and evaluation forms with participants (see details of the data collection in table 1).

**Table 1:** Framework and data collection for the process evaluation

Component	Definition	Data collection
Recruitment	Success of methods used to approach and recruit participants	Parent self-reported questionnaire at baseline: how they heard about the trial
Reach	Degree to which an *intended* audience participates in an intervention	Parent self-reported questionnaire at baseline: sociodemographic characteristics, to define if participants reflect the population Child height and weight measurements
Dose received	Extent of engagement with the FFH and UC interventions by the target population	Parent evaluation questionnaires of each session and end of programme (FFH only) Parent and child one-to-one structured interviews at 3 months (ie, post-intervention)
Dose delivered	The ‘amount’ of intervention provided by the FFH intervention team	Attendance data Facilitators' weekly evaluation forms Facilitators focus groups
Fidelity	The extent to which the FFH intervention was delivered as planned ie, quality and integrity of intervention	Fidelity visits for three to four sessions Facilitators’ focus groups
Perceived impact of intervention	Assessment by intervention participants of the impact of the intervention on themselves	Parent one-to-one structured interviews at 12 months (from baseline)

FFH, Families for Health; UC, usual care.

### Ethics

The National Research Ethics Service Committee West Midlands—Coventry and Warwickshire REC gave ethical approval (reference 11/WM/0290) and participating trusts gave NHS Research and Development approval. A Trial Steering Committee and Data Monitoring and Ethics Committee oversaw the trial. Parents and children gave written informed consent.

Further details of the study design, interventions and outcome measures are available.^14^

## Results

### Participant flow

**Figure 1:**
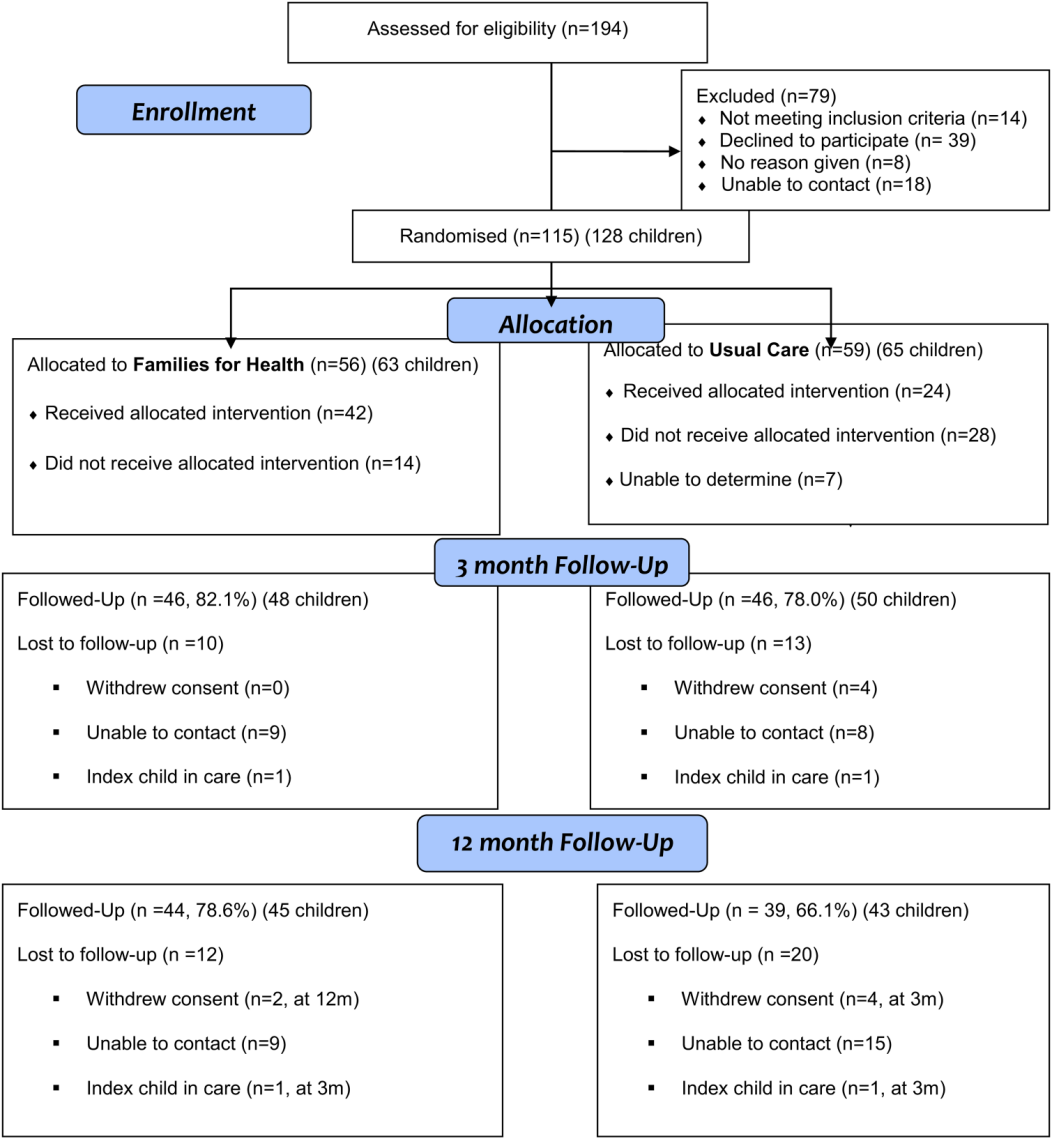
CONSORT flow diagram of Families for Health randomised controlled trial.

We recruited 115 families (with 128 children in total included in the study), with 56 (63 children) allocated to FFH and 59 (65 children) to UC (figure 1). Of the 194 families assessed for eligibility, 79 were excluded (reasons in figure 1). Recruitment took 24 rather than 12 months. Six families withdrew from the trial, four at 3 months and two at 12 months. Reasons for withdrawal were illness, bereavement, child starting secondary school and wanting to manage weight on their own, child disliking study measurements, family feeling the study wasted their time and no reason. Twenty-four families were lost to follow-up for unknown reasons. There was 80% retention of families at 3 months and 72% retention at 12 months, with greater loss to follow-up in the UC arm (figure 1).

### Intervention delivery and attendance

To recruit sufficient families, seven rather than six FFH programmes were delivered. Three FFH programmes ran for 10 weeks as planned and four for 9 weeks (reasons for session cancellations: bad weather (2); shortage of facilitators (1); poor attendance (1)). Minor adaptations were made by facilitators during delivery. Difficulties in delivery related to small group size and broad age range. Of the 56 families randomised to FFH, 21 (37.5%) waited over 3 months to join a group, mostly due to getting a viable number of families together to run the group at a specific site.

Families allocated to FFH were more likely to have attended at least one session than those allocated to UC (42/56, 75.0% vs 24/59, 40.7%; p<0.001) (figure 1). In the FFH arm, 62.5% (35/56) of families completed at least five sessions. FFH was well received by attendees with over 90% of parental ratings of the weekly sessions ‘good’ or ‘great’. Topics receiving the most positive comments were food labelling, coping with stress and building self-esteem.

### Baseline characteristics

With the exception of SES, where the FFH group had higher overall status than the UC group, baseline characteristics were similar (table 2). Eleven families had two or more children participating in the trial. Children from ethnic minority families, single parent families and families where parents were unemployed were a higher proportion of the children recruited than would have been expected from 2011 census data for the localities.

**Table 2:** Baseline characteristics of participating families and children

	FFH	UC	Total
Families, n (%)	56 (48.7)	59 (51.3)	115
Parents/carers, n (%)	64 (46.7)	73 (53.3)	137
Children, n (%)	63 (49.2)	65 (50.8)	128
BMI z-score, mean (SD)	2.69 (0.67)	2.74 (0.70)	2.71 (0.68)
Obese, n (%)	51 (81.0)	55 (84.6)	106 (82.8)
Overweight, n (%)	12 (19.0)	10 (15.4)	22 (17.2)
Families with ≥2 children in study, n (%)	5 (8.9)	6 (10.2)	11 (9.6)
Gender—girls, n (%)	36 (57.1)	29 (44.6)	65 (50.8)
Child age (years), mean (SD)	9.46 (1.57)	9.43 (1.61)	9.44 (1.59)
Child weight (kg), mean (SD)	52.45 (14.22)	52.41 (14.31)	52.43 (14.21)
Child BMI (kg/m^2^), mean (SD)	25.79 (4.44)	25.93 (4.32)	25.86 (4.36)
Child ethnicity, n (%)			
White	38 (60.3)	41 (63.1)	79 (61.7)
Black	4 (6.3)	6 (9.2)	10 (7.8)
Asian	13 (20.6)	9 (13.8)	22 (17.2)
Chinese	0 (0)	0 (0)	0 (0)
Mixed	7 (11.1)	9 (13.8)	16 (12.5)
Other	1 (1.6)	0 (0)	1 (0.8)
Parent/carer age (years), mean (SD)	39.60 (5.86)	40.59 (8.87)	40.13 (7.60)
Parent/carer BMI (kg/m^2^), mean (SD)	31.88 (7.3)	32.01 (8.15)	31.95 (7.74)
Family type, n (%)			
Two parent family	32 (57.1)	28 (47.5)	60 (52.2)
Single parent (mother)	20 (35.7)	26 (44.1)	46 (40.0)
Single parent (father)	0 (0)	0 (0)	0 (0)
Step-family	3 (5.4)	4 (6.8)	7 (6.1)
Other	1 (1.8)	1 (1.7)	2 (1.7)
Socioeconomic status, n (%)			
Managerial/professional	24 (42.9)	15 (25.4)	39 (33.9)
Intermediate	12 (21.4)	7 (11.9)	19 (16.5)
Routine and manual	13 (23.2)	23 (39.0)	36 (31.3)
Never worked/unemployed	7 (12.5)	14 (23.7)	21 (18.3)
Recruitment method, n (%)			
Active	18 (41.7)	25 (58.1)	43 (37.4)
Passive	38 (52.8)	34 (47.2)	72 (62.6)

BMI, body mass index; FFH, Families for Health; UC, usual care.

### Outcome analysis

Table 3 gives the mean values and sample sizes for both trial arms at each time point.

**Table 3:** Simple means and sample sizes for all outcome measures at all time points

	Baseline				3 months				12 months			
	FFH		UC		FFH		UC		FFH		UC	
	n	Mean (95%CI)	n	Mean (95%CI)	n	Mean (95%CI)	n	Mean (95% CI)	n	Mean (95% CI)	n	Mean (95% CI)
Child BMI z-score	63	2.69 (2.52 to 2.85)	65	2.74 (2.57 to 2.91)	48	2.62 (2.43 to 2.82)	50	2.65 (2.45 to 2.85)	45	2.72 (2.54 to 2.89)	43	2.58 (2.37 to 2.79)
Child waist z-score	63	3.33 (3.17 to 3.49)	65	3.27 (3.09 to 3.44)	48	3.19 (3.00 to 3.37)	50	3.21 (3.02 to 3.40)	45	3.32 (3.18 to 3.47)	43	3.09 (2.87 to 3.31)
Child % body fat	63	36.81 (35.25 to 38.36)	65	37.46 (35.80 to 39.13)	48	36.69 (34.77 to 38.61)	50	36.95(35.20 to 38.69)	45	37.58(35.51 to 39.64)	43	35.71(33.64 to 37.79)
Child BMI	63	25.79 (24.67 to 26.91)	65	25.93 (24.86 to 26.99)	48	26.18 (24.74 to 27.61)	50	25.60(24.40 to 26.81)	45	27.30(25.80 to 28.81)	43	25.82(24.69 to 26.95)
Child waist circumference	63	86.17 (83.24 to 89.09)	65	86.30 (83.35 to 89.24)	48	86.44 (82.63 to 90.24)	50	85.62(82.15 to 89.08)	45	90.36(86.79 to 93.92)	43	86.47(83.13 to 89.81)
Child age	63	9.46 (9.07 to 9.86)	65	9.43 (9.03 to 9.83)	48	10.01 (9.56 to 10.45)	50	9.56 (9.10 to 10.03)	45	10.50 (10.05 to 10.94)	43	10.27 (9.77 to 10.77)
Fruit and vegetableconsumption (child)†	63	2.13 (1.60 to 2.65)	65	2.43 (1.95 to 2.91)	48	2.13 (1.54 to 2.71)	50	2.22 (1.75 to 2.69)	45	2.22 (1.64 to 2.85)	41	2.29 (1.74 to 2.85)
EQ-5D-Y (child reported)												
Visual AnalogueScale score†	63	71.95 (65.91 to 77.99)	65	69.92(64.32 to 75.52)	48	70.72(65.12 to 76.33)	50	76.06(69.96 to 82.16)	45	70.22(64.55 to 75.90)	42	73.93(66.86 to 80.99)
Utility score†	63	0.82 (0.77 to 0.87)	65	0.79 (0.72 to 0.86)	48	0.83 (0.76 to 0.90)	50	0.80 (0.73 to 0.88)	43	0.83 (0.77 to 0.90)	41	0.79 (0.71 to 0.88)
EQ-5D-Y (parent reported)												
Visual Analogue Scale score†	63	75.21 (70.38 to 80.03)	65	74.56(69.48 to 79.63)	46	80.91(76.59 to 85.23)	50	83.32(79.17 to 87.47)	44	80.66(76.24 to 85.08)	42	80.26(75.19 to 85.33)
Utility score†	63	0.85 (0.80 to 0.90)	65	0.84 (0.78 to 0.89)	46	0.86 (0.79 to 0.93)	50	0.84 (0.77 to 0.91)	44	0.88 (0.84 to 0.91)	42	0.87 (0.81 to 0.93)
PedsQL Inventory (child reported)												
Overall score†	63	75.91(72.01 to 79.81)	65	78.07(74.39 to 81.76)	48	79.96(75.74 to 84.18)	50	77.90(73.27 to 82.54)	45	79.93(75.42 to 84.43)	42	79.04(74.13 to 83.95)
Psychosocial score†	63	75.00(70.74 to 79.26)	65	76.97(72.97 to 80.97)	48	79.10(74.70 to 83.50)	50	76.61(71.48 to 81.74)	45	78.85(73.85 to 83.86)	42	79.05(73.86 to 84.24)
Physical score†	63	77.54(73.07 to 82.01)	65	80.19(76.19 to 84.18)	48	81.57(76.90 to 86.23)	50	80.25(75.59 to 84.91)	45	81.94(77.45 to 86.44)	42	79.02(74.00 to 84.04)
PedsQL Inventory (parent reported)												
Overall score†	63	72.30(67.42 to 77.19)	65	70.61(66.58 to 74.64)	47	73.72(67.91 to 79.54)	49	73.83(68.34 to 79.31)	45	76.71(71.18 to 82.25)	42	75.98(71.19 to 80.77)
Psychosocial score†	63	71.19(66.14 to 76.24)	65	69.35(65.47 to 73.24)	47	72.80(66.82 to 78.77)	49	73.77(68.74 to 78.80)	45	75.11(69.14 to 81.08)	42	75.67(71.09 to 80.24)
Physical score†	63	74.45(69.24 to 79.66)	65	72.87(67.19 to 78.56)	47	75.51(68.53 to 82.50)	49	73.93(66.74 to 81.12)	45	79.71(73.68 to 85.74)	42	76.58(69.99 to 83.17)
Habitual activity by accelerometer												
MVPA (min/day)†	27	46.96(40.16 to 53.77)	19	52.60(40.22 to 64.98)					27	43.71(36.58 to 50.85)	19	57.81(46.82 to 68.81)
Sedentary (min/day)	27	454.41(425.09 to 483.72)	19	436.68(400.52 to 472.84)					27	458.65(433.90 to 483.40)	19	427.62(384.72 to 470.52)
Step count†	27	8297.94(7552.43 to 9043.46)	19	8316.76(7214.66 to 9418.87)					27	8520.24 (7554.70 to 9485.79)	19	9129.62 (7789.40 to 10469.85)
Parenting style												
Authoritative score†	56	4.08 (3.92 to 4.23)	59	4.02 (3.84 to 4.21)	45	4.13 (3.97 to 4.29)	45	4.04 (3.82 to 4.26)	44	4.19 (4.04 to 4.34)	38	3.97 (3.74 to 4.21)
Authoritarian score	56	1.70 (1.58 to 1.83)	59	1.58 (1.47 to 1.69)	45	1.60 (1.46 to 1.74)	45	1.56 (1.45 to 1.66)	44	1.59 (1.45 to 1.72)	38	1.58 (1.46 to 1.69)
Permissive score	56	2.34 (2.14 to 2.55)	59	2.28 (2.07 to 2.50)	45	2.21 (1.98 to 2.43)	45	2.19 (1.96 to 2.43)	44	2.11 (1.90 to 2.32)	38	2.09 (1.86 to 2.33)
Child-parent relationship												
Overall score†	63	3.93 (3.77 to 4.09)	65	4.11 (3.95 to 4.27)	47	4.05 (3.85 to 4.24)	50	4.18 (3.98 to 4.37)	45	3.98 (3.77 to 4.20)	42	4.12 (3.93 to 4.31)
Conflicts score†	63	3.44 (3.22 to 3.66)	65	3.80 (3.57 to 4.03)	47	3.63 (3.36 to 3.90)	50	3.81 (3.54 to 4.08)	45	3.54 (3.23 to 3.85)	42	3.76 (3.49 to 4.03)
Closeness score†	63	4.49 (4.36 to 4.62)	65	4.47 (4.30 to 4.63)	47	4.53 (4.39 to 4.67)	50	4.60 (4.45 to 4.75)	45	4.49 (4.34 to 4.64)	42	4.53 (4.36 to 4.71)
FEAHQ children subscales*												
Activity level	63	8.16 (3.96 to 12.37)	65	9.09 (5.59 to 12.59)	47	6.91 (3.18 to 10.64)	49	3.79 (0.44 to 7.14)	45	7.09 (2.56 to 11.61)	42	4.73 (-0.42 to 9.87)
Stimulus	63	17.33(14.68 to 19.99)	65	17.26(14.52 to 20.00)	47	14.45(11.90 to 17.00)	49	16.08(12.68 to 19.48)	45	13.78(11.94 to 15.61)	42	15.79(12.74 to 18.84)
Eating related to hunger	63	5.67 (5.19 to 6.14)	65	5.58 (5.17 to 6.00)	47	5.21 (4.67 to 5.76)	49	5.57 (5.07 to 6.08)	45	5.36 (4.83 to 5.88)	42	5.48 (4.91 to 6.04)
Eating style	63	16.43(14.83 to 18.02)	65	14.37(13.03 to 15.71)	47	13.85(12.35 to 15.35)	49	12.90(11.54 to 14.26)	45	14.42(12.62 to 16.22)	42	13.31(11.59 to 15.03)
Parent BMI	64	31.88(30.03 to 33.73)	73	32.01(30.08 to 33.94)	49	30.20(30.04 to 33.96)	49	31.07(29.11 to 33.03)	50	31.82(29.92 to 33.72)	42	29.66(27.67 to 31.64)
Parent % body fat	64	38.48(36.11 to 40.85)	73	39.21(37.10 to 41.32)	47	39.52(37.27 to 41.79)	49	38.72(36.22 to 41.21)	46	38.36(36.05 to 40.67)	41	36.89(34.06 to 39.72)
Parent WEMWBS†	56	47.86(45.48 to 50.23)	57	47.74(45.17 to 50.31)	45	50.00(47.47 to 52.53)	43	48.09(44.78 to 51.40)	44	50.68(47.59 to 53.78)	38	44.95(41.14 to 48.75)

*FEAHQ parent subscales not shown, all between-group differences non-significant.

†Denotes variables where higher scores are better (for other variables lower scores are better).

BMI, body mass index; EQ-5D-Y, EuroQol Five Dimensions Questionnaire Youth Version; FFH, Families for Health; FEAHQ, Family Eating and Activity Habits Questionnaire; MVPA, moderate and vigorous physical activity; UC, usual care; WEMWBS, Warwick-Edinburgh Mental Well-Being Scale.

#### BMI z-score and other anthropometric measures

At 3-month follow-up, within-group analysis showed that the mean BMI z-score was not statistically different from baseline for UC (−0.042, 95% CI −0.089 to 0.004) or FFH (−0.019, 95% CI −0.093 to 0.054), with no difference between trial arms (p=0.593).

**Figure 2:**
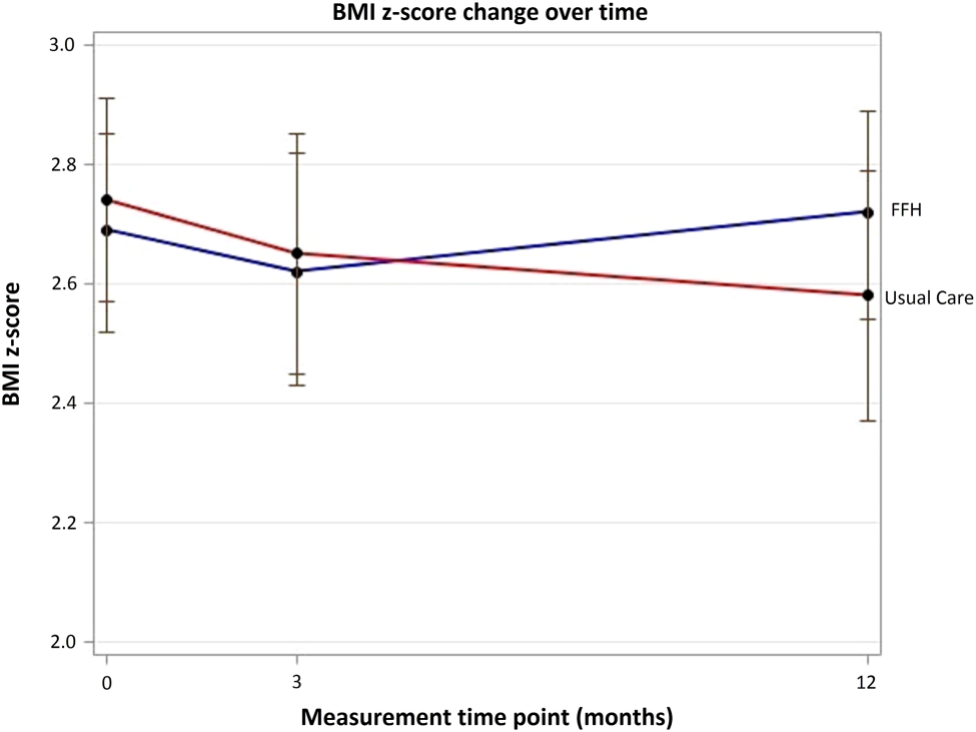
Change over time for primary outcome body mass index (BMI) z-score (unadjusted means and CIs). FFH, Families for Health; UC, usual care.

There was also no difference between trial arms in mean change in BMI z-score from baseline to 12 months, using unadjusted and adjusted analyses (adjusted: 0.114, 95% CI −0.001 to 0.229, p=0.053) (table 4 and figure 2). Within-group analysis showed that BMI z-score was significantly reduced in the UC arm at 12 months (−0.118, 95% CI −0.203 to −0.034, p=0.007), with no change in the FFH arm (−0.005, 95% CI −0.085 to 0.078, p=0.907). The multiple imputation analysis yielded a very similar estimated treatment difference with a significantly greater reduction in BMI z-score in the UC arm than in the FFH arm (0.113, p=0.026). There was also a significantly greater reduction in BMI z-score in the UC arm (0.134, 95% CI 0.008 to 0.259, p=0.037) in an unplanned secondary analysis adjusting for ethnicity and SES in addition to baseline outcome, gender and locality. Results for the change in children's waist z-score were significantly different between groups in favour of UC, although the change in per cent body fat was not significantly different (table 4). Parent's BMI and per cent body fat showed no significant change (table 4).

**Table 4:** Anthropometric measures: between-group differences of changes from baseline (the scores are FFH intervention minus the UC control group)

	Change baseline—3 months		Change baseline—12 months	
	Mean difference (95% CI)	p Value	Mean difference (95% CI)	p Value
BMI z-score (primary)	0.02 (−0.07 to 0.11)	0.633	0.11 (−0.00 to 0.23)	0.053
BMI z-score (unadjusted)	0.02 (−0.06 to 0.11)	0.593	0.11 (−0.00 to 0.23)	0.052
BMI z-score (multiple imputation)			0.113	0.026
Waist z-score	−0.06 (−0.16 to 0.04)	0.211	0.15 (0.00 to 0.29)	0.045
% body fat	0.29 (−0.61 to 1.18)	0.524	1.54 (−0.03 to 3.12)	0.055
Physical activity by accelerometer				
MVPA (min/day)*			−8.46 (−19.37 to 2.45)	0.125
Sedentary (min/day)			13.31 (−35.48 to 62.09)	0.611
Step count*			−591 (−1993 to 811)	0.400
Parent BMI	−0.04 (−0.48 to 0.41)	0.872	−0.08 (−0.97 to 0.81)	0.858
Parent % body fat	0.58 (−0.30 to 1.45)	0.197	0.18 (−1.10 to 1.46)	0.779

*Denotes a positive result favours FFH intervention (for all other variables a negative result favours FFH intervention).

BMI, body mass index; FFH, Families for Health; MVPA, moderate and vigorous physical activity; UC, usual care.

Per-protocol analysis on families that participated in five or more sessions of FFH (‘programme completers’) showed that the mean reduction in BMI z-score was greater in the non-completers (−0.103, 95% CI −0.234 to 0.029) than the completers (0.065, 95% CI −0.040 to 0.169) at 12-month follow-up, although this difference was not statistically different (p=0.059).

#### Behavioural outcomes

We collected 71 pairs (baseline and 12 months) of accelerometer data and 46 records met the inclusion criteria (27 in the FFH arm, 19 in the UC arm). There were no significant differences between groups (table 4).

There was no difference between groups in child-reported fruit and vegetable consumption (table 5). There were no between-group differences at follow-up for the four subscales of the FEAHQ, although there was a significant improvement in activity level in the UC group from baseline, at both 3 months (−5.640, 95% CI −9.985 to −1.295) and 12 months (−6.813, 95% CI −11.176 to −2.450).

**Table 5:** Questionnaire data: between-group differences of changes from baseline (the scores are FFH intervention minus the UC control group)

	Change baseline—3 months		Change baseline—12 months	
	Mean difference (95% CI)	p Value	Mean difference (95% CI)	p Value
Fruit and vegetable consumption*	−0.20 (−1.13 to 0.74)	0.681	−0.25 (−1.25 to 0.75)	0.620
EQ-5D-Y (child reported)				
VAS score*	−7.09 (−17.47 to 3.28)	0.178	−4.37 (−14.58 to 5.85)	0.398
Utility*	0.00 (−0.09 to 0.10)	0.967	0.02 (−0.08 to 0.13)	0.667
EQ-5D-Y (parent reported)				
VAS score*	−7.14 (−15.06 to 0.78)	0.077	−6.60 (−14.75 to 1.56)	0.111
Utility*	−0.02 (−0.10 to 0.06)	0.628	−0.04 (−0.11 to 0.04)	0.332
PedsQL				
Overall (child reported)*	3.71 (−0.31 to 7.73)	0.070	1.88 (−3.65 to 7.41)	0.502
Psychosocial score (child reported)*	3.74 (−0.66 to 8.14)	0.095	0.75 (−5.55 to 7.04)	0.813
Physical (child reported)*	3.86 (−1.51 to 9.24)	0.157	4.20 (−2.63 to 11.02)	0.225
Overall (parent reported)*	−1.60 (−8.22 to 5.11)	0.637	−0.62 (−8.05 to 6.81)	0.868
Psychosocial score (parent reported)*	−2.42 (−8.73 to 3.90)	0.449	−2.09 (−8.97 to 4.80)	0.548
Physical (parent reported)*	−0.02 (−9.40 to 9.38)	0.997	2.17 (−8.69 to 13.03)	0.697
Parenting style				
Authoritative*	−0.05 (−0.27 to 0.16)	0.633	0.03 (−0.18 to 0.25)	0.756
Authoritarian	−0.06 (−0.22 to 0.09)	0.431	−0.10 (−0.26 to 0.06)	0.204
Permissive	−0.02 (−0.29 to 0.25)	0.884	−0.06 (−0.34 to 0.22)	0.684
Child-parent relationship				
Overall*	0.05 (−0.12 to 0.22)	0.569	0.033 (−0.14 to 0.21)	0.714
Conflicts*	0.19 (−0.08 to 0.46)	0.161	0.15 (−0.12 to 0.43)	0.267
Closeness*	−0.11 (−0.32 to 0.11)	0.331	−0.09 (−0.31 to 0.13)	0.405
FEAHQ (child)				
Activity level	4.36 (−1.86 to 10.57)	0.167	4.22 (−2.55 to 10.99)	0.218
Stimulus	1.08 (−3.34 to 5.50)	0.627	0.88 (−3.12 to 4.88)	0.662
Eating/hunger	−0.55 (−1.36 to 0.26)	0.178	−0.32 (−1.03 to 0.39)	0.370
Eating style	−0.66 (−2.52 to 1.20)	0.483	−0.51 (−2.70 to 1.69)	0.648
WEMWBS (parent)*	0.67 (−3.56 to 4.90)	0.754	4.46 (−0.47 to 9.39)	0.076

*Denotes a positive result favours FFH intervention (for all other variables a negative result favours FFH intervention).

EQ-5D-Y, EuroQol Five Dimensions Questionnaire Youth Version; FFH, Families for Health; FEAHQ, Family Eating and Activity Habits Questionnaire; PedsQL, Pediatric Quality-of-Life Inventory; UC, usual care; WEMWBS, Warwick-Edinburgh Mental Well-Being Scale.

#### Psychosocial

There was no significant difference in change from baseline in children's health-related quality of life (assessed by PedsQL and EQ-5D-Y) or in parental mental well-being (table 5). Most parents scored highest on authoritative (desirable) parenting style at baseline and changes in parenting did not differ between groups (tables 3 and 5). The CPRS also showed no difference between (or within) groups (table 5).

### Economic evaluation

**Figure 3:**
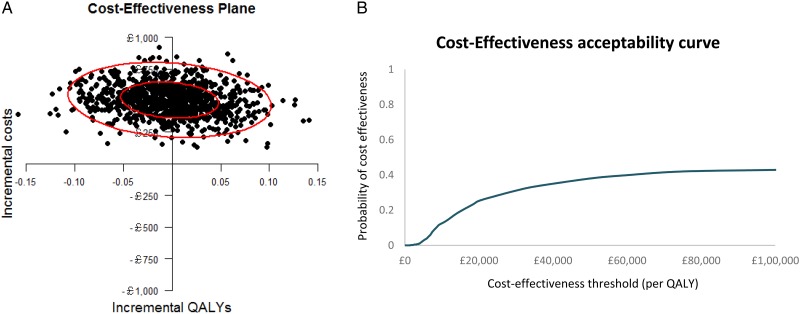
Cost-effectiveness plane (A) and cost-effectiveness acceptability curve (B) for quality-adjusted life-year gained (QALY) outcome; complete cases from the National Health Service and PSS perspective.

Mean (SE) total NHS and personal social service costs over the follow-up period were estimated at £998 (£72) for the FFH group compared with £548 (£73) for the UC group: the cost difference was £450 (bootstrap 95% CI £249, £650; p<0.001). Among children with complete costs and QALY data over the trial follow-up period, FFH was associated with a mean incremental cost of £512 and mean incremental QALYs gained of 0.0009, generating an ICER of £552 175 per QALY gained, far exceeding accepted cost-effectiveness thresholds for an additional QALY.^29^ The cost-effectiveness acceptability curve in figure 3 indicates that, regardless of the value of the cost-effectiveness threshold, the probability that the FFH programme is cost-effective does not exceed 40%. If decision-makers are willing to pay £20 000 for an additional QALY, the probability that the FFH programme is cost-effective is approximately 28%. The economic evaluation remained robust to the choice of study perspective, expression of cost-effectiveness and sensitivity and subgroup analyses exploring the impacts of uncertainty and heterogeneity (see web appendix 1).

### Process evaluation: evidence of change

From coded parent interview responses at 12 months, to a question about what they had changed in their daily lives as a result of their intervention, we were able to categorise the 47 families who participated in this interview into the following: those who had made no change, those who had made change but this was not sustained, those who made at least one change in either food or exercise (no families) food or exercise (no families reported making only changes in parenting) and those who had made multiple changes (see table 6). There was no clear distinction between the FFH and UC groups in terms of count of families and types of changes they reported.

**Table 6:** Changes made (or not) in response to interventions (FFH and control) as reported at 12 months at parental interview

Category of type of change related to the intervention		Control group Number of families Illustrative quote	Intervention group Number of families Illustrative quote
No change made related to the intervention		0	2 “It didn't work out… The children don't like the healthy, that is also hard, and the time, it's really for healthy foods you need more time. It's quicker to put on the fries or to call for something, that is the problem.” Parent 110A
Change not sustained		5 “[Child] goes to my mum's quite a bit, so there's chocolates and my mum bakes so there's lot of things…I mean we're also guilty and we need as parents to lead by example, so both (other parent] and I have perhaps slipped a little bit like crisps have started coming back in the house, there's been chocolate in the fridge, the sugar filled drinks have, you know.” Parent 40A	8 “What I find hard is sustaining it because I don't know… I presume it's normal but since we finished what, 9 months ago did we finish, there's been no reinforcement of it, so we started really well and it's just kind of petered off.” Parent 29A
Single changes	Food change	6 “I want my little one to eat more fruit, so I encourage him. He's very…he has always been so happy when he gets a bag of crisps or a bag of chocolate, but then now when I buy him like fruit or something he's happy as well.” Parent 100A	5 Portion size, definitely changed that. He (the child] still has a little plate anyway. He still has plastic plates, that hasn't changed because once you put food on a big plate you automatically fill the plate.” Parent 109A
	Exercise	4 “I usually park about ten minutes away from the school and he will walk that ten minutes.” Parent 49A	0
	Parenting	0	0
Multiple changes	Parenting-food change	2 “[Child] has like low fat snacks and natural yoghurts and stuff like that in his lunchbox now. He doesn't have major fattening stuff anymore. I don't buy big chocolate bars and things like I did. That ‘who's in charge of what children eat’ was a tough one because it's trying to get him to realise that if I say no I mean no.” Parent 72A	2 “So last night we had lamb chops and salad. It's everything is cooked fresh here and it was never a problem of that, [Child ]'s overeating with the bread and trying to get comfort from sort of the bread. Now she will eat her meals, she will eat her school meal or her sandwiches for school, her breakfast in the morning, and then we don't really pick again after that. There's no asking for toast, we just go through to tea, then bedtime.” Parent 75A
	Multiple food changes	6 “They drink lots more water now. The majority I buy is water now and they will drink things like that whereas before they never used to… Like sweet things are more of a treat now than the norm. I am not just constantly buying that. The shopping's changed, I look at the more healthier options and I am looking at how many calories is in something whereas before I didn't. They taught us how to read the back of labels and what is in them, the calories thing cos I never used to know how to work that out. So that has changed. I just go for more of the healthier options now. Like [Child A]'s lunch box is different, it has got loads of fruit in it and things like that.” Parent 33A	8 “Things get left in the saucepan and if you want more, it is get up and go and get some rather than put it all out on the plate to start off with…I do look at food labelling, a lot, a lot. I must have gone on about this so much at families for health, I have done shopping online now for probably about 18 months, but I have more time now. And so you sit and think ‘well do I want that one?’ so I do look a l lot more.” Parent 10A
	Exercise and multiple food changes	1 “We started karate. We started that about six months ago now. We have all been doing that. I bought a bike so we are doing a bit more cycling… I have been getting healthier food in. Things like yogurts…Breakfast bars instead of sweets and chocolates.” Parent 36A	3 “[Child ] joined the gym, because they went in on a taster sessions and enjoyed it …she's been going three times a week, yes. She quite enjoys it… We use the side plates because the side plates are actually quite big. Because she always has to have seconds… so we give her a small portion at first and then she can have seconds… There's no biscuits in the house, and we only buy now like healthy… the healthier crisps… We don't buy the rubbish now, none of the sweets.” Parent 61A

## Discussion

FFH is designed to increase parenting skills, support family lifestyle change and help children manage their weight. This trial found no difference between trial arms in the change in BMI z-score at 12 months and within-group analysis hinted at the possibility that children in the UC group did better than those allocated to FFH. This was despite higher attendance at FFH than UC, and the FFH programme being rated highly by parents. There was no clear distinction between groups in the changes reported at 12 months by parents from interview data.

FFH was significantly more costly than UC to deliver, mainly because it required separate groups for parents and children, which UC did not. The results of the economic analysis indicate that FFH is unlikely to be cost-effective.

The FFH pilot results^7^ ^8^ were not replicated in this trial, and consideration of the reasons is warranted. Other trials of children's lifestyle interventions have also failed to replicate pilot successes (eg, Kipping *et al*^33^ and Croker *et al*^34^). The difficulties encountered during delivery were not more than would be expected if delivering such an intervention routinely. It may be that scaling up the intervention attenuated its effects. In the pilot study, the intervention was delivered by a small team of four experienced facilitators at one site, and so there may have been some reduction in facilitator skills in the RCT when run at scale across three sites by 17 facilitators mostly new to delivering the programme. Additionally, when evaluating the programme in the context of an RCT, a proportion of families had to wait for a viable group to form because one-half of the families were allocated to the control arm, whereas this did not happen in the pre-post pilot. Alternatively, the pilot study may have been a chance false-positive result.

The parenting components of FFH were based on the Family Links Nurturing Programme (FLNP).^13^ A trial of FLNP was completed during the FFH trial,^35^ finding no evidence of effectiveness as a universal programme. Other parenting interventions for obesity show mixed results. In an RCT of Lifestyle Triple P (Positive Parenting Programme) with children aged 4–11 years in Australia, BMI z-score reduced by −0.11 at the end of the 12-week programme and −0.19 at 1-year follow-up, in contrast to a reduction of −0.01 for the waiting list control.^36^ However, a Dutch study with children aged 4–8 years showed no significant differences in children's BMI z-score between Lifestyle Triple P and the control at 4 and 12 months.^37^ The value of a parenting component to family-based childhood obesity interventions may depend on the baseline parenting styles in trial families. In our study trial, families had good baseline parenting skills, so there was less room for improvement. Families in the trial were also more from ethnic communities, single parents and where parents were unemployed, compared with census data for the localities, which potentially could have impacted on effectiveness and generalisability of the findings to other geographical areas.

The limitations of the study need to be explored to provide lessons learnt for future research, and specifically for RCTs of complex interventions. The pre-post pilot maybe was an inadequate study design to inform the decision to proceed to a full-scale trial. Instead, a pilot RCT would have highlighted potential difficulties with recruitment and with running the intervention in a trial setting, and would have required further thought about the control arm. The decision to compare FFH with ‘UC’ across three sites within the West Midlands meant that the control arm had very varied interventions, which had been developed and improved to suit the needs of their local communities during the time taken to obtain funding and set up the trial. In effect, we did not have a ‘no treatment’ control group. The length of time it took to recruit 115 families (five less than the target) to the trial was grossly underestimated. Only a third of trials recruit the target number within the time specified and around a third have extensions,^38^ and so this issue is not unique to our study. Campbell *et al*^38^ identified that three factors were observed in trials that recruited successfully: having a dedicated trial manager, being a cancer or drug trial and having interventions that were only available within the trial. In the current study, we did have a trial administrator, but the UC interventions were available outside of the trial setting, and of course this is a lifestyle intervention. Future research needs to factor in more realistic recruitment targets, and incorporate further strategies to improve recruitment.

We conclude that FFH is neither effective nor cost-effective for the management of obesity. This trial does not support local or national implementation of FFH.
