# Supplementary material for: Randomised controlled trial and economic evaluation of the ‘Families for Health’ programme to reduce obesity in children
Source: Arch Dis Child. 2016 Dec 21;102(5):416–26. doi: 10.1136/archdischild-2016-311514 (PMC6234231; doi:10.1136/archdischild-2016-311514)
Supplement: supplementary web appendix [file archdischild-2016-311514supp001.pdf]

**Web appendix 1: Economic evaluation**

Methods

*Estimation of resource use and costs*

A comprehensive strategy was adopted to estimate the incremental costs associated with the 'Families for Health' programme. Firstly, the cost of delivering the 'Families for Health' and usual care programmes was assessed, which included the costs of programme development, training of facilitators, staff-related expenses, and revenue and capital overheads. These costs were subsequently converted into programme-specific estimates of cost per session per attending child using separately-collected attendance data. Secondly, data were also collected about all significant health and personal social service and broader societal resource inputs over the 12 month time horizon of the study, between randomisation and 12-months follow-up. The main parent was asked to complete, via researcher administered interviews, a modified version of the Client Services Receipt Inventory (CSRI) to record hospital and community health and social services received by each child, as well as broader service utilisation including educational support, family expenditures and parental lost productivity attributable to the child's health status, at baseline, 3 and 12-months.

Resource inputs were valued using a combination of primary research, based on established accounting methods, and data collated from secondary national tariff sets (Curtis, 2013; Department of Health, 2014) (GB£, 2013-14 prices). Inpatient admissions over this time horizon were delineated by type and duration and valued using per diem costs extracted from the NHS Reference Costs Trusts schedule 2013/14 (Department of Health, 2014). Use of other hospital based care was valued by applying unit costs extracted from national tariffs (Curtis, 2013). Costs for the community based services were calculated by applying unit costs from national tariffs (Curtis, 2013) to resource volumes. NHS net prices per milligram for the medications were obtained from the British National Formulary for Children (BNFC) (Paediatric Formulary Committee, 2013). Costs for individual children were estimated based on their reported doses and frequencies if these were available, or otherwise on an assumed daily dose based on BNFC (Paediatric Formulary Committee, 2013) recommendations. The costs to parents of taking time off work to care for the child(ren) were estimated by applying gender-specific median earnings data (ONS 2012) to occupational classifications derived

from self-reported work status information. Other family-borne costs were valued using data reported by the parents as part of the follow-up resource use questionnaires. Unit costs were inflated where necessary to 2013-14 prices (£ sterling) using the National Health Service Hospital and Community Health Services Pay and Prices Index. No discounting of costs or benefits was applied as the time horizon was less than 12 months.

*Calculation of utilities and quality adjusted life years*

The economic evaluation made use of quality-adjusted life-years (QALYs) to measure preference-based health outcomes. The health-related quality of life of the study children was assessed using the EuroQol EQ-5D-Y (EuroQol) (Eidt-Koch et al., 2009; Wille and Ravens-Sieberer, 2006) obtained from both parents and children at baseline, and 3 and 12 months after randomisation. The standard UK (York A1) tariff values (Dolan 1997) were applied to these responses at each time point to obtain health utility scores. Quality-adjusted life-years (QALYs) were calculated using linear interpolation between baseline and follow-up utility scores and form the main health outcome measure of the economic evaluation.

*Analyses of resource use, costs and outcome data*

Resource use items were summarised by trial allocation group and follow-up period and differences between groups were analysed using t-tests for continuous variables and $\chi^2$ test for categorical variables. Mean (standard error (SE)) costs by cost category and mean (SE) total costs were estimated by trial allocation group for all time periods. Total costs were estimated from both an NHS and personal social services (PSS) perspective and from a broader societal perspective. Cost comparisons were carried out using Student *t* tests. Differences in mean total costs and their respective CIs were estimated. Non-parametric bootstrap (NICE, 2013) estimates based on 1000 replications were also calculated for these differences in mean costs and their respective CIs calculated.

*Cost-effectiveness analyses*

The main cost-effectiveness analyses were conducted for complete cases (i.e. those with complete cost and outcome data). The cost-effectiveness results were expressed primarily in terms of an incremental cost-effectiveness ratio (ICER). This was calculated as the difference in mean costs divided by the difference in mean outcomes (QALYs or change in BMI-z score between baseline and 12-months) between the trial comparators. The primary analyses took the perspective of the NHS and Personal Social Services. The nonparametric bootstrapping approach was used to determine the level of sampling uncertainty surrounding the mean ICER by generating 10,000 estimates of incremental costs and benefits. These were represented graphically on four quadrant cost-effectiveness planes. Cost-effectiveness acceptability curves (CEACs) showing the probability that the 'Families for Health' programme was cost-effective relative to usual care across a range of cost-effectiveness thresholds were also generated based on the proportion of bootstrap replicates with positive incremental net benefits. Unless otherwise stated, all statements about cost effectiveness are based on a £20,000 per QALY gained threshold. The probability that 'Families for Health' is less costly or more effective than usual care was based on the proportion of bootstrap replicates that had negative incremental costs or positive incremental health benefits.

Secondary analyses were also conducted where the outcomes remained unchanged from the main cost-effectiveness analyses but for the costs a wider societal perspective was taken that included broader economic costs.

*Sensitivity and sub-group analyses*

Several sensitivity analyses were undertaken to assess the impact of areas of uncertainty surrounding components of the economic evaluation. These involved re-estimating the main cost-effectiveness outcomes under the following scenarios: 1) conducting a per protocol analysis where families having participated in 5 or more sessions of the 'Families for Health' programme are regarded as 'programme completers', i.e. as having complied with the protocol sufficiently; 2) multiple imputation of all missing cost and outcomes data; 3) parent-reported EQ-5D-Y values for the study child(ren) substituted for child self-reported values in the formulation for QALYs; and 4) incorporation of EQ-5D values reflecting the main parent's self-reported health within calculations of overall QALYs gained.

Sub-group analyses were conducted for the main cost-effectiveness results to explore heterogeneity in the trial population. These were conducted by: (i) age group (6-8 years, 9-11 years); (ii) gender (boys, girls); and (iii) site (Site A, Site B, Site C).

## Results

### *Cost-effectiveness*

When a study perspective of the NHS and personal social services was adopted (i.e. that adopted for the baseline analysis) and health outcomes were measured in terms of QALYs, the average total cost was £1,019 in the 'Families for Health' group, compared with £507 in the usual care group, generating a mean incremental cost of £512 (among those with complete cost and QALY data),. The mean incremental cost-effectiveness of the 'Families for Health' programme was estimated at £552,175 per QALY gained. The cost-effectiveness acceptability curve shown in Figure 3 of the main body of the paper indicates that regardless of the value of the cost-effectiveness threshold, the probability that the 'Families for Health' programme is cost-effective does not exceed 40%. If decision-makers are willing to pay £20,000 for an additional QALY, the probability that the 'Families for Health' programme is cost effective is approximately 28%. Broadening the study perspective to that of society as a whole had little effect on these cost-effectiveness results. In particular, the mean incremental cost-effectiveness ratio remained relatively static at £559,115 per QALY gained and the probability that the 'Families for Health' programme is cost effective at a £20,000 cost-effectiveness threshold remained unchanged at 28%.

When a study perspective of the NHS and personal social services was adopted and health outcomes were measured in terms of longitudinal change in BMI z score, the average total cost was £998 in the 'Families for Health' group, compared with £548 in the usual care group, generating a mean incremental cost of £450 (among those with complete cost and BMI z score data). The mean incremental cost-effectiveness of the 'Families for Health' programme was estimated at -£3935 per unit change in BMI z score. Regardless of the value of the cost-effectiveness threshold, the probability that the 'Families for Health' programme is cost-effective did not exceed 2% (on the basis of the BMI z score). If decision-makers are willing to pay £20,000 per unit change in BMI z score, the probability that the 'Families for Health' programme is cost effective was less than 1%. Broadening the study perspective to that of society as a whole had little effect on these cost-effectiveness results. In particular, the

mean incremental cost-effectiveness ratio remained relatively static at -£3,748 per unit change in BMI z score and the probability that the 'Families for Health' programme is cost effective at a £20,000 cost-effectiveness threshold remained unchanged at less than 1%.

*Sensitivity analyses*

Several sensitivity analyses were undertaken to assess the impact of uncertainty on the cost-effectiveness results. A 'per protocol' analysis was performed that defined 'programme completers' as families that participated in 5 or more sessions of the 'Families for Health' programme and non-completers as families that participated in less than 5 sessions of the 'Families for Health' programme. These analyses were also restricted to the baseline NHS and personal social services perspective. Of particular note is that for programme completers, the mean incremental cost per QALY gained attributable to the 'Families for Health' programme declined to £27,790 and the probability that the programme is cost effective at a £20,000 cost-effectiveness threshold increased to 43%. This compared to a mean incremental cost per QALY gained for the programme of -£6,441 (indicating that usual care is dominant in health economic terms) and a probability that the programme is cost effective at a £20,000 cost-effectiveness threshold of 17% amongst non-completers.

We also re-estimated cost-effectiveness following multiple imputation of all missing cost and outcomes data, i.e. estimates were calculated for all 128 study children. Of particular note is that following multiple imputation, the mean incremental cost per QALY gained (assuming a NHS and personal social services perspective) attributable to the 'Families for Health' programme declined to £9119 and the probability that the programme is cost effective at a £20,000 cost-effectiveness threshold increased to 67%. Similar results were observed when a societal perspective for costs was adopted. In contrast, when the BMI z score was considered as the health outcome measure, the 'Families for Health' programme remained dominated by usual care in health economic terms and the probability that the programme is cost-effective did not exceed 6% regardless of the value of the cost-effectiveness threshold.

We also re-estimated cost-effectiveness when alternative sources and inputs for EQ-5D utility values were incorporated into the analyses. Using parent-reported EQ-5D-Y values for the study child(ren) to substitute for child self-reported values in the formulation for QALYs removed the incremental QALY benefit associated with the 'Families for Health'

programme. The probability that the programme is cost effective at a £20,000 cost-effectiveness threshold declined to 23% when a NHS and personal social services perspective was adopted and 25% when a societal perspective was adopted. Furthermore, the reduction in incremental QALYs associated with the ‘Families for Health’ programme increased when EQ-5D values reflecting the main parent’s self-reported health were also incorporated within calculations of overall QALYs gained. The probability that the programme is cost effective at a £20,000 cost-effectiveness threshold declined to 2%.

*Sub-group analyses*

Several sub-group analysis were conducted to explore the heterogeneity in our cost-effectiveness results. The sub-groups considered in our analyses were: (i) age group (6-8 years, 9-11 years); (ii) gender (boys, girls); and (iii) site (Site A, Site B, Site C). The cost-effectiveness results for two particular sub-groups of children are worthy of comment. First, when the QALY measure was considered as the primary health outcome measure, the probability of cost-effectiveness of the ‘Families for Health’ programme was notably higher for girls than for boys: 67% versus 15% at a £20,000 cost-effectiveness threshold from a NHS and personal social services perspective and 63% versus 16% at a £20,000 cost-effectiveness threshold from a societal perspective. Second, when the QALY measure was considered as the primary health outcome measure, the probability of cost-effectiveness of the ‘Families for Health’ programme was notably higher in Site A than in Site B or Site C: 61% versus 11% versus 36% at a £20,000 cost-effectiveness threshold from a NHS and personal social services perspective and 64% versus 7% versus 35% at a £20,000 cost-effectiveness threshold from a societal perspective. These patterns were not replicated for the BMI z-score outcome measure.

Web Appendix References

Curtis L. Unit costs of Health and Social Care 2013. Kent: Personal Social Services Research Unit, University of Kent; 2013.

Department of Health. NHS Reference Costs 2013–2014. National Schedule of Reference Costs. London: Department of Health; 2014.

Dolan P. Modeling Valuations for EuroQol Health States. Medical care. 1997;35(11):1095-108.

Eidt-Koch D, Mittendorf T, Greiner W. Cross-sectional validity of the EQ-5D-Y as a generic health outcome instrument in children and adolescents with cystic fibrosis in Germany. BMC pediatrics. 2009;9(1):55.

National Institute for Health and Care Excellence, (NICE). Guide to the methods of technology appraisal. NICE: Londo, UK; 2013.

Office for National Statistics. Annual Survey of Hours and Earnings. London: Office for National Statistics; 2012.

Paediatric Formulary Committee. British National Formulary for Children. (No 66). London: BMJ Group, Pharmaceutical Press, and RCPCH Publications; 2013.

Wille N, Ravens-Sieberer U, editors. Age-appropriateness of the EQ-5D adult and child-friendly version–Testing the feasibility, reliability and validity in children and adolescents. 23rd Scientific Plenary Meeting of the EuroQol Group in Barcelona, Spain: September 14; 2006.
